# Gender Differences in Familiar Face Recognition and the Influence of Sociocultural Gender Inequality

**DOI:** 10.1038/s41598-019-54074-5

**Published:** 2019-11-29

**Authors:** Maruti V. Mishra, Jirapat Likitlersuang, Jeremy B Wilmer, Sarah Cohan, Laura Germine, Joseph M. DeGutis

**Affiliations:** 1 Department of Psychiatry, Harvard Medical school, Boston, MA USA; 2 Boston Attention and Learning Laboratory, VA Boston Healthcare, Jamaica Plain Division, 150 S Huntington Ave., Boston, MA USA; 3 Department of Psychology, Wellesley College, 106 Central Street, Wellesley, MA 02481 USA; 4 Harvard Medical School, Harvard University, 401 Park Drive, Suite, 504W Cambridge, MA USA; 5 Institute for Technology in Psychiatry, McLean Hospital, Belmont, MA USA

**Keywords:** Human behaviour, Social behaviour

## Abstract

Are gender differences in face recognition influenced by familiarity and socio-cultural factors? Previous studies have reported gender differences in processing *unfamiliar* faces, consistently finding a female advantage and a female own-gender bias. However, researchers have recently highlighted that unfamiliar faces are processed less efficiently than familiar faces, which have more robust, invariant representations. To-date, no study has examined whether gender differences exist for *familiar* face recognition. The current study addressed this by using a famous faces task in a large, web-based sample of  > 2000 participants across different countries. We also sought to examine if differences varied by socio-cultural gender equality within countries. When examining raw accuracy as well when controlling for fame, the results demonstrated that there were no participant gender differences in overall famous face accuracy, in contrast to studies of unfamiliar faces. There was also a consistent own-gender bias in male but not female participants. In countries with low gender equality, including the USA, females showed significantly better recognition of famous female faces compared to male participants, whereas this difference was abolished in high gender equality countries. Together, this suggests that gender differences in recognizing unfamiliar faces can be attenuated when there is enough face learning and that sociocultural gender equality can drive gender differences in familiar face recognition.

## Introduction

Gender differences in cognitive performance and its origins have important implications for models of cognitive abilities as well as society. Consistent gender differences have been reported in visuospatial tasks such as mental rotation^1^, visual working memory^2^, visual motion processing^3^, sustained attention^4^, emotion recognition^5^, face recognition^6^, and episodic memory recollection^7^, with females showing superior performance over males in most of the tasks except for visuospatial attention tasks where males perform better than females. Though it is debated whether these differences are driven by biological or socio-cultural factors^8,9^, many studies emphasize the impact of the latter^10–14^. The aims of the current study were twofold; first, we sought to understand gender differences in face recognition beyond *“unfamiliar”* face recognition (the rapid learning of previously unfamiliar faces) to *“familiar”* face recognition (recognizing faces that one has semantic knowledge about and previous exposure). Second, we used a large, multi-country sample to probe for any modulation of gender differences by socio-cultural gender equality.

Previous studies on gender differences in face processing have focused on the perception and recognition of unfamiliar faces. These differences were observed specifically in within-task learning and recognition paradigms^15–17^ or simultaneous perceptual matching paradigms^6,18,19^, with females showing better performance than males. Further, superior recognition of unfamiliar faces in females has shown to be highly robust and invariant to face view^20^, gaze direction^21^, face-race^22,23^ as well as duration of presentation^15,24^. Studies have also reported own-gender biases, with females being consistently better at recognizing female than male faces^6,24,25^ and less consistently reported a male own-gender bias^26,27^. These effects were also supported by multiple eye movement^28^ and electrophysiological studies^26,29,30^. Notably, two recent studies suggest that female superiority in face recognition can be reduced when there is sufficient face learning^31^ or prior experience^32^ with faces or face categories used. For example, Heisz *et al*.^31^, conducted a four-day face recognition study for unfamiliar faces, where faces were repeated each day, and showed that the female advantage in response accuracies on the first day was eliminated on the fourth day with repeated face learning.

Despite the extensive literature on gender differences in learning and recognizing *unfamiliar* faces, no study to date has closely examined gender differences in recognizing *familiar* faces. Though unfamiliar face stimuli are easier to manipulate and control in laboratory settings, in real-world situations we are typically required to identify familiar faces that are learned over many instances and for whom detailed semantic knowledge is available. Because of this enhanced learning, familiar faces have shown to be processed more efficiently than unfamiliar faces, reflected by faster, and more accurate recognition^33–35^. For example, severe image degradation and image distortion has very little effect on the ability to recognize familiar faces, whereas this severely disrupts recognizing unfamiliar faces^36,37^.

To study the role of familiarity in face recognition, a common approach has been to recall the identity of famous faces. The recollection of semantic (e.g., name, profession) and/or episodic information required by these tasks is quite different from typical matching and recognition tasks used for unfamiliar faces. In particular, most unfamiliar face recognition tasks do not present semantic information along with the face (though see Sperling *et al*.^38^) and recognition judgments may rely more on ‘familiarity’, i.e., feeling of knowing, rather than recollecting specific contextual and semantic details^39–41^. Further, the extent or degree of familiarity is also dependent on frequency of prior exposure and subsequent learning. Previous famous faces recognition studies^42–44^ have not reported or examined gender differences. Famous face recognition has shown to involve distinct processing from unfamiliar faces^34,45^, including extended face learning through repeated exposure, acquiring semantic and episodic knowledge associated with the face, and more reliance on recollection than familiarity^39,46^. Given these processing differences between unfamiliar and familiar faces, it is essential to understand to what extent previous theories supporting female superiority in unfamiliar face recognition are generalizable and influenced by face learning and familiarity.

Socio-cultural factors such as ethnicity and in-group/out-group effects have also shown to influence face processing, but there have been limited investigations on how they contribute to gender differences in face recognition performance^47^. Previous studies have examined how socio-cultural gender equality affects gender differences in mathematics performance^48^, episodic memory^10^, and attention^4^. Further, it is also reported that these differences depend on the degree of gender equality, existing at the country level^4,11,14,48^. For example, Riley *et al*.^4^ reported greater gender differences in sustained attentional control in countries with low gender equality, in comparison to countries with high equality. Notably, these effects were driven primarily by changes across countries in female rather than male participants. Whether and how socio-cultural factors influence gender differences for familiar face recognition has not been addressed previously and was one of the motivations of the current study.

To answer these questions, we used a web-based online study that allowed us to measure face recognition across a large sample spanning different countries. Given the potential influence of the degree of fame of celebrities (fame scores) on recognition accuracy, we also examined accuracy after regressing out fame. Further, previous studies have reported an episodic and recognition memory advantage in females in general^31,49^ and specifically in unfamiliar face recognition^22^, which may predict a female advantage at recollecting semantic information associated with famous faces. Thus, based on studies of unfamiliar face recognition tasks and the female advantage in episodic memory, we expected to observe gender differences in famous face recognition, with superior performance for females in overall face recognition ability and an own-gender bias that is stronger for females than for male participants. Considering that gender equality across countries relates to cognitive abilities and strategic biases^4,11,12,14^, we also sought to explore whether different levels of gender equality in countries would moderate gender differences in famous face recognition. Based on previous studies^4^, we hypothesized that greater gender equality in a country would be associated with reduced gender differences and reduced own-gender biases in famous face recognition. Alternatively, it is also possible that since the famous faces are highly familiar, we might not observe gender differences^31^ or a gender-based interaction.

## General Methods

### Participants

Participants voluntarily visited the TestMyBrain.org website (https://testmybrain.org/), that was openly available for anyone, during 2014–2015. A total of 2,770 participants were included in this study (age range = 18–50 years). For each analysis section below, we provide the separate details about participants’ age and gender. Before starting the test, participants provided online informed consent in English, irrespective of their language or country of origin. Each volunteer was given a unique electronic ID and had a unique IP address of the computer from which they ran the task, that was recorded to identify the country where the task was performed. Participants were provided with individualized feedback after completing the task. All the experiments performed here followed the guidelines approved by the Institutional Ethics Committee on the Use of Human Subjects at Harvard University. TestMyBrain.org is a citizen science website that people can visit voluntarily to participate in a variety of neurocognitive tasks in exchange for personalized feedback. Data from TestMyBrain.org has been shown to be of comparable high quality and reliability when compared with data gathered in a laboratory setting^50^ and has been extensively used to study population dynamics across various cognitive, perceptual and neuropsychological tests and experiments^4,51–53^.

### Stimuli and Design

The stimuli consisted of 69 front-view faces of famous celebrities taken from google images advanced searches (publicly available and free to use, share or modify as described in the usage rights at the google image advanced search database, under the CC-BY-SA-3.0 license, https://creativecommons.org/licenses/by-sa/3.0/) that were included in three famous face tests (FFMT1–27 faces, FFMT2–40 faces, FFMT3–26 faces). The faces were cropped to remove extra facial features like hair, ears and area below the jawline. This study was designed as a web-based study that can be run on a PC/Desktop/iPad/mobile phone. For small screen devices the participants were instructed to rotate the screen to the maximum display width. In accordance with this, the size of face image was made to scale up/down depending on the size of the screen used, maintaining the aspect ratio, and thus keeping the image size constant. The visual angle for all the face images were ~ 5.5° × 7°. The faces belonged to people from various professions including actors/actresses, politicians, musicians and sports personalities (for the list of faces used please refer to Table S1, Supplementary materials). An independent t-test for age of the faces, calculated using each celebrity’s date of birth, showed that males (n = 43, *M* age = 56.23, *SD* = 15.77) were significantly older than females (n = 26, *M* age = 42.35, *SD* = 14.66) (*t*(67) = 3.64, *p* < 0.001, Cohen’s *d* = 0.90).

### Procedure

**Figure 1:**
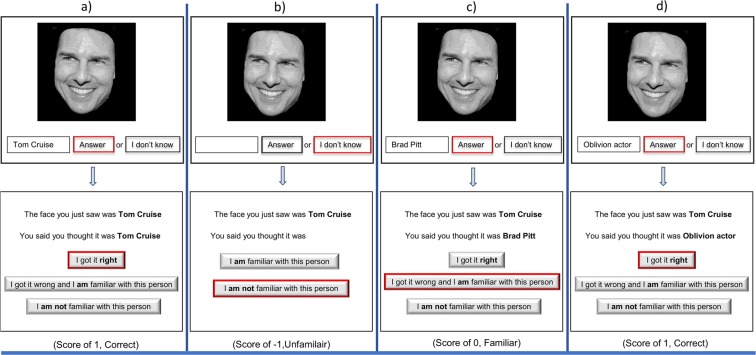
*Trial structure for famous faces recognition task*. Example of four types (**a–d**) of possible trial structure from the experiment. The possible choices made by the participant are highlighted with red box. (**a**) A single face (representative image) is shown at the center of the screen (first row) with a response box and two choices. Once they respond, the second screen (second row) displays the choices. Once they select the required option, the next image (**b**), first row) is displayed on the screen. The task was self-paced. (The modified image was adapted from https://commons.wikimedia.org/wiki/File:Tom_Cruise_avp_2014_4.jpg, available under CC-BY-SA-3.0 license, https://creativecommons.org/licenses/by-sa/3.0/).

For each face presented at the center of the screen (Fig. 1), participants were asked to make their best guess about the identity of the person by typing in the box provided and click ‘submit’, or to click a button that said “I don’t know”. For example, if the face shown was of Tom Cruise, and they could not remember the name but typed that he was the “Top Gun actor” OR “actor Cruise”, they were instructed to self-score their responses as correct (Fig. 1d). After they entered a response, the correct answer/name of the person was displayed on the screen and they were prompted to click on either of the following: “*I got it Right*”; “*I got it wrong and I am familiar with this person*”; or “*I got it wrong and I am not familiar with this person*”. If the participant did not enter a guess but left the answer field blank (Fig. 1b) and chose the option “*I do not know”*, they were then provided with the answer on the next screen and asked to choose from either “*I am familiar with this person*” OR “*I am not familiar with this person*”. The experiment took approximately 10–15 minutes. There were three famous face tests (FFMT1–27 faces, FFMT2–40 faces, FFMT3–26 faces) and test assignment was randomized across participants. Each test has the faces presented only once. The task across the three version of the test was identical. Each version of the test included a different subset from a total pool of 69 faces, with 24 faces co-occurring in two versions of the test but never repeated in any test or within a participant. This co-occurrence was due primarily to one test (FFMT1) being an earlier version of another test (FFMT2). There were no significant differences in either fame scores (*p* = 0.44) or accuracy (*p* = 0.33) between stimuli that co-occurred in two tests versus one test. After the task, participants were asked to provide demographic information, such as ethnicity and education. Then, feedback was provided about their performance on the test.

## Analysis approach

### Data preprocessing

Given that the data were obtained based on participants' self-score, before analysis, we screened the data for three types of erroneous trials, where: 1) a correct answer was typed but the participant scored themselves as being ‘incorrect’, 2) an incorrect answer was typed and they scored themselves as being ‘correct’, and 3) there was no response typed but still the participant scored themselves as ‘correct’. In all such cases, if more than 50% of the trials showed this pattern in any participant, the entire case was removed from further analysis (2.71% of participants). For those showing less than 50% of such trials, we eliminated these trials (3% of trials) rather than the participants. As we were interested in assessing participants’ face recognition performance on only faces that they had exposure to and were familiar with, we removed face items where participants indicated they were ‘*not familiar with this person’*. Removal of these trials is consistent with numerous studies that have used famous faces to diagnose face recognition deficits^55^. The total percentage of trials used (only familiar trials) in each test is provided in the supplementary Fig. S7. During prescreening of the raw data, we did not consider spelling or typing errors as a rejection criterion. We favor the above self-scoring method as it allows misspellings of the correct answer to be scored as correct, and thus produce accurate face recognition responses^54^.

### Data processing

#### Statistical analysis

To increase power, we collapsed the data across the three famous face tests. Before doing so, we confirmed that the three tests did not significantly differ in the participant gender x face gender interactions (3-way interaction *F*(1, 2122) = 0.84, *p* = 0.43). Considering this, we collapsed the data across tests and our main statistical analysis approach was to do a two-way mixed ANOVA, with the participant gender as a between-subject variable and the face gender as a within-subject variable. Statistical significance was examined at alpha = 0.05 significance level. As we were interested in comparing participants’ responses on male and female faces separately, Bonferroni corrected planned comparisons were performed for any significant interaction effect. Effect sizes such as using partial eta squared values (η_p_^2^) and Cohen’s *d* are reported for *F*-tests and *t*-tests, respectively. These analyses were executed using an open source statistical software JASP 0.9.2.0 (https://jasp-stats.org/) and planned comparisons were done using online tool of GraphPad posttest calculator (https://www.graphpad.com/quickcalcs/).

#### Using fame normalization approach

It is plausible that famous people, whose faces are used in this study, would have differences in frequency of exposure in media, or that famous males might be generally more famous than famous females due to certain sociocultural factors. Additionally, we also found that the famous males were significantly older in age than famous females, suggesting that participants may have had more exposure to famous males than females. In order to account for bias arising from either of these factors, apart from the standard raw accuracy scores statistical analysis, we also sought to calculate accuracy after controlling for fame.

Recently, using sophisticated computational meta-analysis^56^, various famous figures in history have been ranked and given fame scores, that have been successfully applied that use large datasets^57–59^. Using the fame score for the celebrities that were used in our study, first we tested for fame differences and found that the fame scores were significantly different for famous males and females. An independent t-test showed that famous males (*M* = 5.51, *SD* = 0.99) had significant higher fame scores (*t* (67) = 2.66, *p* = 0.010, *d* = 0.66), than famous females (*M* = 4.89, *SD* = 0.82). Further, we also report that the fame scores and age of the celebrities correlated significantly (*r* = 0.33), suggesting that older celebrities are more famous. It should be noted that after regressing out fame, there is still leftover variation in how distinctive the face is, how typical the image is of the person, etc. that could explain variability in accuracy.

We normalized for fame by calculating the residual scores For this, we correlated the identification accuracy of famous faces with their fame scores (Supplementary Table S1), where in the identification accuracy correlated significantly (Supplementary Figs. S1–S3, scatter plots (c) & (d)). The mean accuracy score for each face (the scores for any duplicate faces were averaged) across all the three tests were plotted as a function of fame scores^56^, and the resulting linear regression equation (Fig. S1(d)) was used to calculate the predicted score for each individual participant. This is because each participant showed variability in face categorization accuracy. For each participant, the fame scores were used for only those faces that were either correctly recognized (score of ‘1’) or familiar (score of ‘0’). These were then separately averaged based on gender of the faces, to get a separate gender-based fame normalized predicted values. Later, the average response for male and female faces from each participant were subtracted from the predicted values to get the residual scores for the two face genders, that we used in statistical analysis. By this, we attempted to remove the effect of fame from the accuracy scores. This normalization was separately done for the different country groups, with their resulting face identification accuracy scores. Again, for reference purposes, we also report the fame normalized analysis for all the trials (familiar and unfamiliar), that is not a part of our main analysis, in the supplementary section (Figs. S1–S3, bar plot (f)).

#### Additional supplementary analyses

Though we did not use the trials whenever the respective famous faces were unfamiliar to any of the participants, for reference purposes we report the raw analyses using all the face trials, irrespective of whether familiar or unfamiliar to each participant, in the supplementary materials (see Figs. S1–S3, (e) & (f) bar plots). We have also provided the percentage of trials used in each test for each country to calculate face recognition accuracy (Fig. S7). We also provide the original data and graphs for proportion of familiar faces (that is used in the main analysis to calculate proportion of correct responses) and unfamiliar faces for all the trials, for each gender of the face and participant gender (Supplementary Figs. S1–S3, bar plots (a) & (b)) along with the distribution of individual participant response scores (Supplementary Figs. S4–S6) for all three countries using sinaplots in R software.

## Results

### Analysis 1: Gender differences in famous face recognition among the USA sample

Our primary objective here was to understand whether there are gender differences and own-gender biases in recognition of male and female famous faces. We selected USA adults 18–50 years old because most of the faces were US celebrities and because 18–50 years old is when face recognition is typically at its best^60^.

#### Participants

A total of 2,295 USA adults (Age range = 18 to 50 years) were included in this analysis. After exclusions using data preprocessing, and no responses in any trials, the total number of analyzed subjects was 2,128 (FFMT1–238 Males and 494 females, FFMT2–255 males and 466 females, FFMT3–217 males and 458 females). Overall there were 710 males (*M* age = 29.49, *SD* = 9.24) and 1,418 females (*M* age = 29.49, *SD* = 9.76) with very similar age distributions.

#### Results

**Figure 2:**
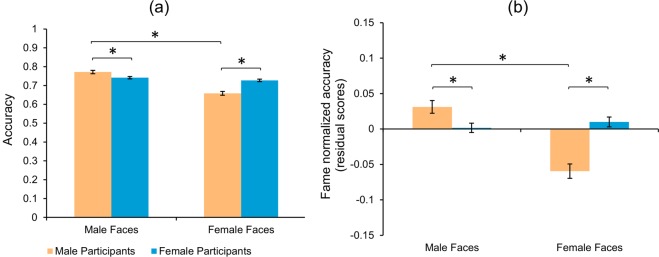
*USA face recognition accuracy*. Bar plot of accuracy scores for famous faces. (**a**) raw scores (**b**) fame normalized values. Error bars represent standard error of mean. *p < 0.05.

A two-way mixed ANOVA on face recognition accuracy for participant gender and famous face gender (Fig. 2a, Table 1) showed a main effect of face gender, *F*(1, 2126) = 238.4, *p* < 0.001, η_p_^2^ = 0.10, where male famous faces were recognized more accurately than female faces. Participant gender showed no main effect, *F* (1, 2126) = 3.17, *p* = 0.08; but there was a significant participant gender x face gender interaction, *F* (1, 2126) = 142.0, *p* < 0.001, η_p_^2^ = 0.06. Importantly, for famous female faces, planned comparisons showed that female participants performed significantly better (mean difference = 0.07, 95% CI [−0.08, −0.054], *t*(2126) = 11.87, *d* = 0.26) than males. Conversely, male participants performed significantly better (mean difference = 0.03, 95% CI [0.015, 0.045], *t*(2126) = 5.16, *d* = 0.124) in recognizing famous male faces. Further, we calculated an own-gender bias in male participants by subtracting face recognition performance obtained for male faces vs. female faces and in a similar way for female participants (female vs. male faces performance). A significant own-gender bias was observed only for male participants (mean difference = 0.114, 95% CI [0.097, 0.13], *t*(2126) = 16.98, *d* = 0.45); but not for female participants.

**Table 1:** Mean values for all 3 countries.

Countries	Face Gender	Participant gender	Familiar trials mean values (proportions)			Residual mean values			n
			Mean	SD	SEM	Mean	SD	SEM	
USA	Male faces	Male	0.772	0.237	0.009	0.031	0.238	0.009	710
		Female	0.742	0.244	0.006	0.002	0.246	0.007	1418
	Female faces	Male	0.658	0.271	0.010	−0.060	0.270	0.010	710
		Female	0.727	0.258	0.007	0.010	0.258	0.007	1418
High Gender Equality	Male faces	Male	0.678	0.280	0.032	0.069	0.281	0.032	76
		Female	0.614	0.266	0.026	0.003	0.268	0.026	107
	Female faces	Male	0.516	0.324	0.037	−0.067	0.325	0.037	76
		Female	0.543	0.322	0.031	−0.036	0.323	0.031	107
Low Gender Equality	Male faces	Male	0.607	0.296	0.025	0.068	0.294	0.025	143
		Female	0.549	0.341	0.036	0.018	0.344	0.036	91
	Female faces	Male	0.499	0.351	0.029	0.001	0.352	0.029	143
		Female	0.589	0.367	0.038	0.095	0.368	0.039	91

The table displays mean values for raw scores and residual scores used in main analysis. (M = mean, SD = standard deviation, SEM = standard error of mean, n = Participant sample).

Fame normalization (see Methods section, Fig. 2b, Supplementary Fig. S1d) showed similar results. In particular, a two-way mixed ANOVA found a main effect for face gender, *F*(1, 2126) = 98.70, *p* < 0.001, η_p_^2^ = 0.044, a significant interaction, *F*(1, 2126) = 142.67, *p* < 0.001, η_p_^2^ = 0.063; but no difference in participant gender, *F*(1, 2126) = 3.38, *p* = 0.07. Post-hoc comparisons (Table 1) revealed that female participants had significantly better recognition accuracy for famous female faces (mean difference = −0.07, 95% CI [−0.085, −0.06], *t*(2126) = 12.04, *d* = 0.27) compared to male participants. In contrast, male participants showed significantly better recognition accuracy for male famous faces (mean difference = 0.03, 95% CI [0.014, 0.044], *t*(2126) = 4.99, *d* = 0.12), though this effect was smaller in magnitude. Again, a significant own-gender bias was shown for male participants (mean difference = 0.09, 95% CI [0.074, 0.108], *t*(2126) = 13.56, *d* = 0.36) but not for female participants (mean difference = −0.008, 95% CI [−0.020, 0.004], *t*(2126) = 1.68).

*Result summary.* In contrast to findings of unfamiliar face recognition^6,24^, our results demonstrated no significant overall accuracy differences between male and female participants and a significant own-gender advantage in only *male* participants. We did find an accuracy advantage for recognizing famous male vs. female faces, even after regressing out fame. Interestingly, the male vs. female participant difference was much larger (Cohen’s *d* = 0.26) when recognizing female famous faces, compared to the male vs. female difference when recognizing male famous faces (Cohen’s *d* = 0.12).

In the next set of analyses, we sought to examine potential cultural effects on these gender differences; focusing on countries with greater gender equality (Analysis 2a) and less gender equality (Analysis 2b) compared to the USA sample. The objective criteria to select the relevant countries in the two groups were decided based on a previous study conducted in our laboratory^4^. We were interested to know whether the greater male vs. female participant difference for female faces compared to male faces was due to cognitive biases arising because of moderate gender inequality in the USA. In other words, it could be that cultural/institutional gender inequality in America (e.g., males are in more positions of power than females) could have led male participants to be biased to ‘attend-to’ and ‘individuate’ male faces more than female faces, resulting in reduced performance on female famous faces.

### Analysis 2: Does the gender based difference depend on gender inequality existing in different cultural societies?

We wanted to explore whether cultural factors were influencing the observed gender differences in famous face recognition performance. In order to address this, we compared the results of countries that show either higher levels of gender equality than the USA (e.g., Norway, Finland, Sweden, Denmark and Netherlands, according to the United Nations Gender Inequality index (GII), http://hdr.undp.org/en/content/gender-inequality-index-gii) or countries that show lower gender equality than the USA (e.g., India, Pakistan, Brazil, Egypt, and Indonesia). The higher the GII index, the higher the gender inequality in those countries. The average gender inequality ratio in the year 2014–15, across the three country groups was 0.05 for high gender equality countries, 0.21 for the USA and 0.49 for low gender equality countries. We hypothesized that we would observe reduced male vs. female participant accuracy differences and reduced own-gender biases in countries with higher sociocultural gender equality while greater gender differences in accuracy and greater own-gender biases in countries that show reduced gender equality.

### Analysis 2a: Countries with high gender equality

#### Participants

We selected the Scandinavian countries (Sweden (n = 59), Denmark (n = 20), Netherlands (n = 43), Norway (n = 39) and Finland (n = 25) from our dataset that had previously shown to have the highest gender equality^4^. We grouped the data from these five countries to achieve enough power to detect effect sizes similar to the USA analysis. A total of 203 Scandinavian adults (18–50 years) were included in this analysis. Post data screening, there were 183 participants (FFMT1–24 males and 35 females, FFMT2–23 males and 37 females, FFMT3–29 males and 35 females) with a total of 76 males (*M* age = 30.88, *SD* = 7.91) and 107 females (*M* age = 30.96, *SD* = 9.67) having similar ages. To calculate the fame-normalized residual scores, we used the exact same procedure as in previous analysis except that we used the regression equation (Fig. S2(d)) from the Scandinavian dataset predicting accuracy from fame scores. This was because the fame-to-accuracy relationship may be slightly different for Scandinavian countries due to less exposure to American celebrities than the USA sample.

#### Results

**Figure 3:**
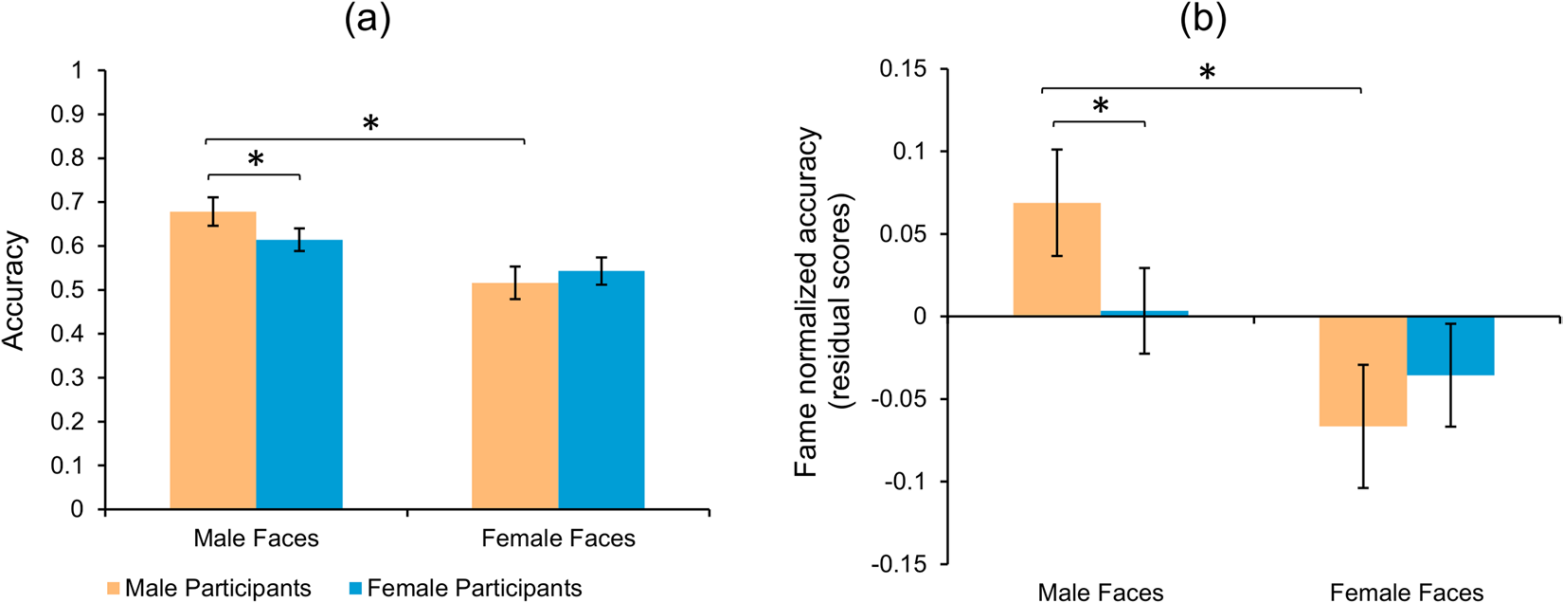
*High gender equality countries face recognition accuracy.* Bar plot of accuracy scores for (**a**) raw famous face accuracy and (**b**) fame-normalized accuracy. Error bars represent standard error of the mean. *p < 0.05.

A two-way mixed ANOVA for accuracy (Fig. 3a) showed a main effect of face stimulus gender, *F*(1, 181) = 63.02, *p* < 0.001, η_p_^2^ = 0.26, where famous male faces were recognized more accurately than famous female faces (Table 1). Though we did not find any significant main effect of participant gender, *F*(1, 181) = 0.193, *p* = 0.66, there was a significant interaction, *F*(1, 181) = 9.68, *p* = 0.002, η_p_^2^ = 0.05. In contrast to the USA sample, there was ***no*** significant difference (mean difference = −0.027, 95% CI [−0.07, 0.02], *t*(181) = 1.31) between the male and female participants in recognizing female faces. However, for famous male faces, a significant difference was observed (mean difference = 0.064, 95% CI [0.02, 0.12], *t*(181) = 3.10, *d* = 0.23), between male and female participants. We also observed a significant own-gender bias for male participants (mean difference = 0.162, 95% CI [0.11, 0.22], *t*(181) = 7.245, *d* = 0.54), with males performing better at male faces than female faces; but no own-gender bias for female participants.

Normalizing for fame did not change the results (Fig. 3b, Table 1). That is, there was a main effect of face gender, *F*(1, 181) = 35.99, *p* < 0.001, η_p_^2^ = 0.17, a significant interaction, *F* (1, 181) = 11.00, *p* = 0.001, η_p_^2^ = 0.06; but no difference in participant gender, *F* (1, 181) = 0.16, *p* = 0.69. Importantly, planned comparisons for famous female faces did not show any significant difference (mean difference = −0.03, 95% CI [−0.08, −0.02], *t*(181) = 1.49) between the male and female participants. However, there was a significant difference for famous male faces (mean difference = 0.07, 95% CI [0.02, 0.11], *t*(181) = 3.19, *d* = 0.24) between males and females. There was also a significant own-gender bias only for male participants (mean difference = 0.136, 95% CI [0.08, 0.19], *t*(181) = 6.08, *d* = 0.45) but not for female participants.

#### Result summary

Our analyses of Scandinavian countries that are reported to have very high socio-cultural gender equality, showed a largely similar pattern to the USA sample. There were no overall participant gender differences in accuracy, participants performed better on male than female faces, and there was an own-gender bias only for male participants. Interestingly, in contrast to the USA sample, there was no gender differences in recognizing female famous faces. This suggests that greater gender equality in a country may lead to more similar male and female performance on famous female face recognition. Though it is unclear why there was not a similar reduction in gender differences for famous male faces, we sought to further investigate this difference by examining performance in countries with low gender equality. We predicted that there would be significant differences between male and female participants on female face recognition.

### Analysis 2b: Countries with lowest gender equality

#### Participants

We next selected five countries that had lowest gender equality as reported previously^4^: India (n = 205), Brazil (n = 23), Egypt (n = 10), Pakistan (n = 19), and Indonesia (n = 31) and combined them to provide us with a sufficient sample size. A total 275 adult participants (18–50 years) were included from these countries. After data prescreening, there were 234 participants (143 males and 91 females: FFMT1 = 63 males, 37 females; FFMT2 = 36 males, 25 females; FFMT3 = 44 males, 29 females), with very similar age range (males, *M* = 26.87, *SD* = 7.15; females, *M* = 25.75, *SD* = 6.94).

#### Results

**Figure 4:**
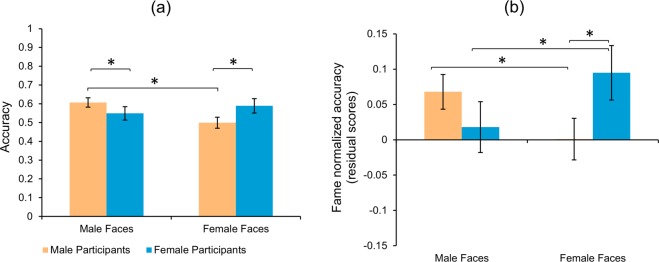
*Low gender equality countries face recognition accuracy*. Bar plot of accuracy scores for famous faces. (**a**) raw scores. (**b**) fame normalized values. Error bars represent standard error of mean. *p < 0.05.

The two-way ANOVA for accuracy responses (Fig. 4a) showed a main effect of face gender, *F*(1, 232) = 5.646, *p* = 0.018, η_p_^2^ = 0.021, where male faces were recognized more accurately (mean difference = 0.05, SE = 0.015, *t* = 3.44, *d* = 0.23, *p* < 0.001) than female faces. There was no main effect of participant gender, *F*(1, 232) = 0.14, *p* = 0.71, but again a significant interaction was observed, *F* (1, 232) = 27.18, *p* < 0.001, η_p_^2^ = 0.11. Planned comparisons showed better performance by females participants in recognizing female faces (mean difference = −0.09, 95% CI [−0.14, −0.04], *t*(232) = 4.53, *d* = 0.25), while males performed significantly better (mean difference = 0.06, 95% CI [0.008, 0.108], *t*(232) = 2.92, *d* = 0.18) at recognizing male faces. Further, a significant own-gender bias was observed for male participants (mean difference = 0.108, 95% CI [0.064, 0.152], *t*(232) = 6.16, *d* = 0.33) but not for female participants.

Normalizing for fame (Figs. 4b, S3.(d), Table 1) did not show a main effect of face gender, *F*(1, 232) = 0.122, *p* = 0.73, or participant gender, *F*(1, 232) = 0.25, *p* = 0.62, but a significant interaction between the two, *F*(1, 232) = 26.138, *p* < 0.001, η_p_^2^ = 0.10. As we predicted, planned comparisons revealed that female participants performed significantly better (mean difference = −0.094, 95% CI [−0.14, −0.04], *t*(232) = 4.73, *d* = 0.26) than males in recognizing famous female faces. No significant difference (mean difference = 0.05, 95% CI [0.00, 0.10], *t*(232) = 2.5) was observed between male and female participants for recognizing male faces. Additionally here, an own-gender bias was observed for both male (mean difference = 0.067, 95% CI [0.023, 0.11], *t*(232) = 3.82, d = 0.21) and female participants (mean difference = −0.077, 95% CI [−0.132, −0.022], *t*(232) = 3.50, d = 0.22).

#### Results summary

Again, the raw accuracy analyses showed similar results to the USA sample. That is, male faces were recognized more accurately than female famous faces and again only males showed an own-gender bias in face recognition. Further, as predicted, countries with lower sociocultural gender equality showed significant and pronounced gender differences in famous female face recognition (*d* = 0.25), similar to the USA (*d* = 0.26) for both raw and fame regressed analyses. This contrasts the results from high gender equality countries where we observed no gender differences in female famous faces recognition.

#### Analysis 3. Comparing Performance between USA, high gender equality, and low gender equality countries

**Figure 5:**
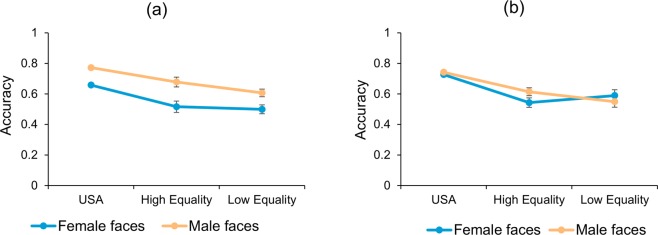
*Cross country comparison for famous face recognition*. Raw accuracy values for familiar trials plot against the country performance for (**a**) male participants (**b**) female participants, separately shows variable face recognition accuracy in females compared to males in different country groups. Error bars represent SEM.

We next sought to determine if the patterns observed across the different countries were significantly different (see Fig. 5). It should be noted that this comparison is not ideal because our main dependent measure of interest—fame-normalized accuracy—cannot be compared directly across cultures due to using separate fame vs. accuracy regression equations. Still, we were able to examine the overall raw accuracy. We first performed a three-way mixed ANOVA, 3 (Country) × 2(Participant gender) × 2(Face gender), showing main effects of face gender, *F*(1, 2539) = 126.99, *p* < 0.001, η_p_^2^ = 0.05 and country, *F*(1, 2539) = 62.454, *p* < 0.001, η_p_^2^ = 0.05, but not participant gender. There was only a trend towards a three-way interaction, *F*(1, 2539) = 1.842, *p* = 0.159, suggesting that overall accuracy pattern across countries did not differ significantly.

Interactions across countries for each participant gender were also examined separately, since previous studies^4,10,12,14,48^ suggested that factors like less employment, gender inequality and loss of equal opportunity for females in any given society affected performance in female participants more than males. Here, the two-way ANOVA, country (3) × face gender (2), for males showed main effect of face gender (own-gender bias), *F*(2, 926) = 176.05, *p* < 0.001, η_p_^2^ = 0.16, but only a trend towards a significant interaction between gender of the faces and countries, *F*(2, 926) = 2.3, *p* = 0.097. On the other hand, the ANOVA with female participants did not show a main effect of face gender, *F*(2, 1613) = 3.14, *p* = 0.076, but did show a significant interaction between face gender and countries, *F*(2, 1613) = 9.801, *p* < 0.001, η_p_^2^ = 0.012. Our results suggest that female participants performed better on male faces than female faces for both USA and Scandinavian countries, while this pattern was reversed for low gender equality countries where female participants showed an own-gender bias (Fig. 5). This suggests that, for familiar face recognition, socio-cultural gender equality particularly affects accuracy in female participants.

## General Discussion

Gender differences in face recognition have previously been reported only with unfamiliar faces, where identity recognition depends on short-term learning and familiarity matching. However, it was unclear if these differences were present for more well-learned familiar/famous faces. Previous studies have also never investigated sociocultural influences (e.g., gender equality in a country) on gender differences in face recognition. We investigated these outstanding questions by having a large web-based sample (*N* > 2000) of participants from countries with differing levels of gender equality perform male and female famous face recognition. Our results show three important findings: a) across all countries there were no overall significant participant gender differences in famous face recognition, and faces of famous males were generally recognized better than famous females, b) we observed significant own-gender biases for male but not female participants, and c) gender equality across countries significantly affected performance on famous female faces, where there was less of a difference between male and female participants in high gender equality countries compared to low gender equality countries. These findings have important implications for models of gender differences in face recognition as well sociocultural effects on cognition.

Contrary to previous studies of unfamiliar faces^27,61^, for familiar face recognition we did not find any evidence of overall accuracy differences between male and female participants. Thus, though past studies show that males generally perform worse than females with unfamiliar faces and may be slower to learn faces, once they learn a face, they are able to identify it as accurately as females. This suggests that specialized mechanisms for efficient, robust identification of familiar faces are equally engaged by males and females. Our findings are consistent with a recent eye-tracking study that used multiple exposures for faces and showed that there was an initial female-over-male participant recognition advantage for recognizing unfamiliar faces that was abolished as faces were learned over a period of four days^31^. Our results extend these findings and show that prior experience and learning reduce gender differences in face recognition. The similar male/female familiar face recognition performance is also consistent with the observation of a similar incidence of developmental prosopagnosia in males and females^62^, which is often diagnosed by deficits in familiar face recognition.

### Why is there an advantage in recognizing famous male vs. famous female faces?

We consistently found, across all countries, that there was an advantage for recognizing famous males vs. females faces. Our findings did not change even after regressing out fame for USA and high gender equality countries, though in low gender equality countries there was no male/female residual accuracy difference. Our results contradict previous findings from unfamiliar face recognition studies^6^. In fact, in one study^24^ where cropped and full unfamiliar faces were used, an advantage for recognizing female faces was observed. This was driven by females being better at female faces, while males performed equally well on male and female faces. One possible explanation could be that, in the current study, famous males had more exposure in media and thus to the participants, leading to effects of prior experience^32,35^ that might account for better identification accuracy. Related to this overall male famous face advantage, we also found that there was a consistent own-gender bias only for male participants but not in females, though both male and female participants showed own-gender biases in low equality countries after regressing out fame. Further, females performed equally well for both male and female faces. These results are again opposite to those observed in unfamiliar face recognition studies that report a stronger own-gender bias in females^6,15,17,22–24,27^. Apart from the fact that the previous studies used unfamiliar faces, an own-gender bias in familiar or famous faces has never been previously reported. Though it is likely that the male own-gender bias in our study was driven by the main effect of participants performing overall better at male famous faces, additional studies would be useful to confirm this finding.

### Does socio-cultural gender inequality modulate gender differences in face recognition?

A novel finding from our study is sociocultural gender equality does affect face recognition, but only for female famous faces. Specifically, we found that, for recognizing famous female faces, male participants were substantially worse than female participants in the USA and lowest gender-equality countries, while there were no participant gender differences in countries with high gender equality. This pattern of results remained even after controlling for fame in each analysis. Interestingly, we did not find an effect of cultural gender equality on male famous face accuracy, with all cultures showing a similar pattern of male participants outperforming female participants. This finding fits with previous research showing that culture can differentially affect cognitive processes^63–65^, where differential performance is observed based on the socio-cultural background of the participants. Further, the cross-country analysis showed that female participants significantly varied in famous face recognition across countries while the performance of males was relatively stable irrespective of the cultural background.

A possible explanation for the observed sociocultural effects is that there are different gender roles in the social structure of societies. For example, in certain countries (such as India, Bangladesh or Egypt) males more often go out and participate in larger social networks while females participate in smaller social networks and are mostly indoors. This may lead to differences in perceptual learning experiences^18,66^. It could also be that in such societies, compared to countries with higher equality, female faces are more outgroup members to males and ingroup to females, which may lead male participants to individuate female faces less than female participants. Though plausible in lower equality countries, this explanation cannot account for the USA results, as both males and females equally participate in large social groups. Another explanation for the cultural effect on female face recognition could be that in lower gender-equality countries and the USA, male participants could be biased to process females in a less individuated manner compared to female participants. Further, it could be that females from lower gender-equality countries individuate famous females more than men, while males from lower gender equality countries have more of a propensity to categorize females rather than individuate them.

It is notable that the female face advantage for female participants is present despite *only* including trials that participants reported to being familiar with. This suggests that all participants had some familiarity with the famous females faces but that, in lower-equality countries and the USA, female participants were better able to recollect individuating information (e.g., name, professional details, etc.) about the faces compared to male participants. This explanation fits with dual process accounts of recognition memory^41^ suggesting that judgments are based on either recollection (the retrieval of contextual and semantic details about an item), or familiarity (the feeling that an item has been experienced previously without retrieval of additional information). Previous studies examining the other-race effect have found that subjects rely more on recollection memory for own-race compared to other-race faces^67^, which could be driven by more effortful and semantic encoding of own-race faces^68^. Similarly, in countries with lower gender equality or the USA, female participants may put forth more effort and semantically encode famous female faces compared to male participants. It is notable that this effect is abolished in high gender-equality countries, suggesting that male and female participants equally encode and retrieve semantic information about famous female faces.

Though these results are intriguing, they would be more convincing if we found a similar effect of cultural gender equality on male famous face accuracy. That said, previous literature on gender differences in cognition have often reported that female participants’ performance mostly drives and impacts gender differences, and is more affected (improved or reduced) by cultural norms like labor force, education, and employment^10,11,69^. Our results are consistent with these studies and extend these female-driven differences to face recognition. Though not often applied to the face literature, several theories have been proposed to explain gender disparity arising due to cultural differences like the gender similarity theory^70^, gender stratification (lack of equal opportunities to both genders) theory^71^, and socio-cultural theory^69^. Together, they suggest that, the greater the difference in power and status between men and women in a culture, the greater would be the gender difference in psychological or cognitive domains (e.g., math performance^48,69^). Indeed, along with our study, a few of the other major studies do show that gender inequality increases the gap in psychological variables such as math performance^48^ and sustained attention^4^, and that it is specific to female participants.

Though the results of the current study are compelling, there are a few limitations. First, even though we focused on the particular faces participants reported being familiar with, we did not account for the differential degree of prior exposure of semantic knowledge for each face. Another limitation is that some of the individuals may be more well-known by their *‘faces’* and other may be better known by their ‘*names’* (e.g., actors vs. musicians or historical figures). Additionally, it is likely that other-race effects reduced accuracy in the low gender-equality countries since most of the faces used were Caucasian. Though it is unclear whether this would bias the results, replicating the study with own-race faces in low equality countries would be useful. We would also like to note that our results are limited to the dichotomous (male vs. female) nature of gender classification rather than considering it as a continuous spectrum.

To conclude, by utilizing a set of famous faces in a large cross-cultural sample, we demonstrate that male and female participants have a similar capacity for familiar face recognition but vary in their attention to and expertise with male and female famous faces. Results from high gender equality countries suggest that, encouragingly, sociocultural context can decrease at least some of these gender differences in face recognition. These results help set the stage for future investigations examining the complex interactions between culture, gender, and cognition.

## Supplementary information

Supplementary File

## Data Availability

The datasets generated during the current study are available from the corresponding authors on request.
