# Supplementary material for: Gender Differences in Familiar Face Recognition and the Influence of Sociocultural Gender Inequality
Source: Sci Rep. 2019 Nov 29;9:17884. doi: 10.1038/s41598-019-54074-5 (PMC6884510; doi:10.1038/s41598-019-54074-5)
Supplement: Supplementary file 1 — Supplementary File [file 41598_2019_54074_MOESM1_ESM.pdf]

Gender Differences in Familiar Face Recognition and the Influence of Sociocultural Gender Inequality

Maruti V. Mishra[1,2], Jirapat Likitlersuang[1,2], Jeremy Wilmer[3], Sarah Cohan[4], Laura Germine[5], Joseph M. DeGutis[1,2 *]

Supplementary materials.

Table S1. List with the Name, Identity, test number and fame scores of the famous faces used in this study

FFMT name	Face Number	Gender	Profession	Actual Name	Identity	Fame-Score	Age
FFMT1	1	Female	Actor/TV	Oprah Winfrey	Oprah Winfrey	5.701	65
FFMT1	2	Male	Actor/TV	George Timothy Clooney	George Clooney	5.446	57
FFMT1	3	Male	Musician	Michael Joseph Jackson	Michael Jackson	7.054	50
FFMT1	4	Male	Actor/TV	John Joseph Nicholson	Jack Nicholson	5.164	81
FFMT1	5	Male	Actor/TV	James Thomas Fallon	Jimmy Fallon	4.457	44
FFMT1	6	Male	Musician	Justin Randall Timberlake	Justin Timberlake	5.365	38
FFMT1	7	Female	Actor/TV	Kristen Jaymes Stewart	Kristen Stewart	4.284	28
FFMT1	8	Male	Actor/TV	John Christopher Depp II	Johnny Depp	5.665	55
FFMT1	9	Male	Actor/TV	William Bradley Pitt	Brad Pitt	5.7	55
FFMT1	10	Female	Actor/TV	Norma Jeane Mortenson (Baker)	Marilyn Monroe	6.15	36
FFMT1	11	Male	Politician	William Jefferson Clinton	Bill Clinton	7.111	72
FFMT1	12	Male	Politician	Martin Luther King, Jr	Martin Luther King	6.378	39
FFMT1	13	Male	Actor/TV	Robin McLaurim Williams	Robin Williams	5.38	63
FFMT1	14	Male	Actor/TV	Conan Christopher O’Brien	Conan O'Brien	5.1938	55
FFMT1	15	Male	Musician	Justin Drew Bieber	Justin Bieber	4.722	25
FFMT1	16	Female	Actor/TV	Hannah Dakota Fanning	Dakota Fanning	4.646	25
FFMT1	17	Male	Actor/TV	Denzel Hayes Washington, Jr.	Denzel Washington	5.054	64
FFMT1	18	Female	Musician	Taylor Alison Swift	Taylor Swift	5.014	29
FFMT1	19	Male	Actor/TV	Jonah Hill Feldstein	Jonah Hill	3.829	35
FFMT1	20	Female	Musician	Demetria Devonne Lovato	Demi Lovato	4.601	26
FFMT1	21	Female	Politician	Michelle LaVaughn Obama	Michelle Obama	4.96	55
FFMT1	22	Female	Musician	Stefani Joanne Angelina Germanotta	Lady Gaga	5.724	32

FFMT1	23	Female	Actor/TV	Kimberly Noel (Kardashian) West	Kim Kardashian	4.557	38
FFMT1	24	Female	Actor/TV	Lucy Alexis Liu	Lucy Liu	4.369	50
FFMT1	25	Female	Actor/TV	Kristen Anne Bell	Kristen Bell	4.124	38
FFMT1	26	Male	Actor/TV	John Joseph Travolta	John Travolta	5.128	65
FFMT1	27	Female	Politician	Hillary Diane Rodham Clinton	Hillary Clinton	6.479	71
FFMT2	1	Male	Politician	Martin Luther King, Jr.	Martin Luther King	6.378	39
FFMT2	2	Male	Actor/TV	William Bradley Pitt	Brad Pitt	5.7	55
FFMT2	3	Male	Politician	George Walker Bush	George Bush, “W”	7.872	72
FFMT2	4	Female	Actor/TV	Kimberly Noel (Kardashian) West	Kim Kardashian	4.557	38
FFMT2	5	Male	Actor/TV	John Joseph Travolta	John Travolta	5.128	65
FFMT2	6	Female	Actor/TV	Kristen Jaymes Stewart	Kristen Stewart	4.284	28
FFMT2	7	Male	Actor/TV	James Douglas Muir Leno	Jay Leno	5.17	68
FFMT2	8	Male	Actor/TV	Daniel Jacob Radcliffe	Daniel Radcliffe, Harry Potter	4.991	29
FFMT2	9	Male	Musician	Elvis Aaron Presley	Elvis Presley	7.019	42
FFMT2	10	Female	Musician	Miley Ray Cyrus	Miley Ray Cyrus	5.529	26
FFMT2	11	Male	Actor/TV	George Timothy Clooney	George Clooney	5.446	57
FFMT2	12	Male	Politician	Barack Hussein Obama II	Barack Obama	7.4	57
FFMT2	13	Female	Actor/TV	Oprah Winfrey	Oprah Winfrey	5.701	65
FFMT2	14	Male	Actor/TV	Mark Robert Michael Wahlberg	Mark Wahlberg, Marky Mark	4.808	47
FFMT2	15	Female	Musician	Stefani Joanne Angelina Germanotta	Lady Gaga	5.724	32
FFMT2	16	Male	Actor/TV	James Thomas Fallon	Jimmy Fallon	4.457	44
FFMT2	17	Female	Actor/TV	Norma Jeane Mortenson (Baker)	Marilyn Monroe	6.15	36
FFMT2	18	Male	Musician	Justin Drew Bieber	Justin Bieber	4.722	25
FFMT2	19	Male	Actor/TV	Robin McLaurim Williams	Robin Williams	5.38	63
FFMT2	20	Male	Actor/TV	David Michael Letterman	David Letterman	5.131	71
FFMT2	21	Female	Musician	Barbara Streisand	Barbara Streisand	5.528	76
FFMT2	22	Male	Politician	William Jefferson Clinton	Bill Clinton	7.111	72

FFMT2	23	Male	Musician	Shawn Corey Carter	JayZ	5.841	49
FFMT2	24	Male	Actor/TV	Robert Douglas Thomas Pattinson	Robert Pattinson	4.202	32
FFMT2	25	Female	Politician	Hillary Diane Rodham Clinton	Hillary Clinton	6.479	71
FFMT2	26	Male	Actor/TV	Arnold Alois Schwarzenegger	Arnold Schwarzenegger	6.147	71
FFMT2	27	Female	Actor/TV	Elizabeth Stamatina Fey	Tina Fey	4.581	48
FFMT2	28	Male	Musician	Michael Joseph Jackson	Michael Jackson	7.054	50
FFMT2	29	Male	Actor/TV	John Joseph Jack Nicholson	Jack Nicholson	6.164	81
FFMT2	30	Female	Musician	Taylor Alison Swift	Taylor Swift	5.014	29
FFMT2	31	Male	Actor/TV	Benjamin Géza Affleck-Boldt	Ben Affleck	5.029	46
FFMT2	32	Male	Actor/TV	Orenthal James Simpson	O. J. Simpson	5.166	71
FFMT2	33	Female	Actor/TV	Lucy Alexis Liu	Lucy Liu	4.369	50
FFMT2	34	Male	Actor/TV	William Henry Cosby, Jr.	Bill Cosby	5.38	81
FFMT2	35	Male	Actor/TV	Denzel Hayes Washington, Jr.	Denzel Washington	5.054	64
FFMT2	36	Male	Actor/TV	David Jude Heywor th Coan Law	Jude Law	4.736	46
FFMT2	37	Male	Actor/TV	Willard Carroll Smith, Jr.	Will Smith	5.432	50
FFMT2	38	Female	Politician	Michelle LaVaughn (Robinson) Obama	Michelle Obama	4.96	55
FFMT2	39	Male	Actor/TV	William James Murray	Bill Murray	2.834	68
FFMT2	40	Male	Musician	Justin Randall Timberlake	Justin Timberlake	5.365	38
FFMT3	1	Male	Politician	John Fitzgerald Kennedy	John F Kennedy, JFK	6.816	46
FFMT3	2	Female	Actor/TV	Angelina Jolie	Angelina Jolie	5.348	43
FFMT3	3	Male	Musician	Sir James Paul McCartney CH MB E	Paul McCartney	6.477	76
FFMT3	4	Female	Actor/TV	Mary-Kate Olsen	Mary Kate Olsen / Ashley Olsen	3.732	32
FFMT3	5	Female	Actor/TV	Tyra Lynne Banks	Tyra Banks	4.787	45
FFMT3	6	Female	Actor/TV	Jennifer Shrader Lawrence	Jennifer Lawrence	2.431	28
FFMT3	7	Female	Musician	Jessica Ann Simpson	Jessica Simpson	5.283	38
FFMT3	8	Female	Politician	Margaret Hilda Tha tcher (Baroness)	Margaret Thatcher	6.277	87

FFMT3	9	Male	Actor/TV	James Eugene Carrey	Jim Carrey	5.11	57
FFMT3	10	Female	Musician	Selena Marie Gomez	Selena Gomez	4.909	26
FFMT3	11	Male	Actor/TV	Thomas Cruise Mapother IV	Tom Cruise	5.632	56
FFMT3	12	Female	Actor/TV	Susan Abigail Sarandon	Susan Sarandon	4.698	72
FFMT3	13	Male	Actor/TV	John Christopher Depp II	Johny Depp	5.665	55
FFMT3	14	Female	Actor/TV	Scarlett Ingrid Johansson	Scarlet Johansson	5.075	34
FFMT3	15	Female	Actor/TV	Julia Fiona Roberts	Julia Roberts	4.947	51
FFMT3	16	Male	Musician	Fred Astaire	Fred Astaire	4.927	88
FFMT3	17	Female	Musician	Madonna Louise Ciccone	Madonna	6.643	60
FFMT3	18	Female	Actor/TV	Nicole Mary Kidman, AC	Nicole Kidman	5.347	51
FFMT3	19	Male	Sports	David Robert Joseph Beckham	David Beckham	5.857	43
FFMT3	20	Female	Actor/TV	Uma Karuna Thurman	Uma Thurman	4.557	48
FFMT3	21	Female	Actor/TV	Demi Gene (Guynes) Kutcher	Demi Moore	4.664	56
FFMT3	22	Male	Actor/TV	Joseph Leonard Gordon-Levitt	Joseph Gordon-Levitt	4.045	38
FFMT3	23	Male	Actor/TV	Thomas Jeffrey Hanks	Tom Hanks	5.574	62
FFMT3	24	Male	Musician	Kanye Omari West	Kanye West	5.841	41
FFMT3	25	Male	Actor/TV	Robert Douglas Thomas Pattinson	Robert Pattison	4.202	32
FFMT3	26	Female	Musician	Miley Ray Cyrus	Miley Ray Cyrus	5.529	26

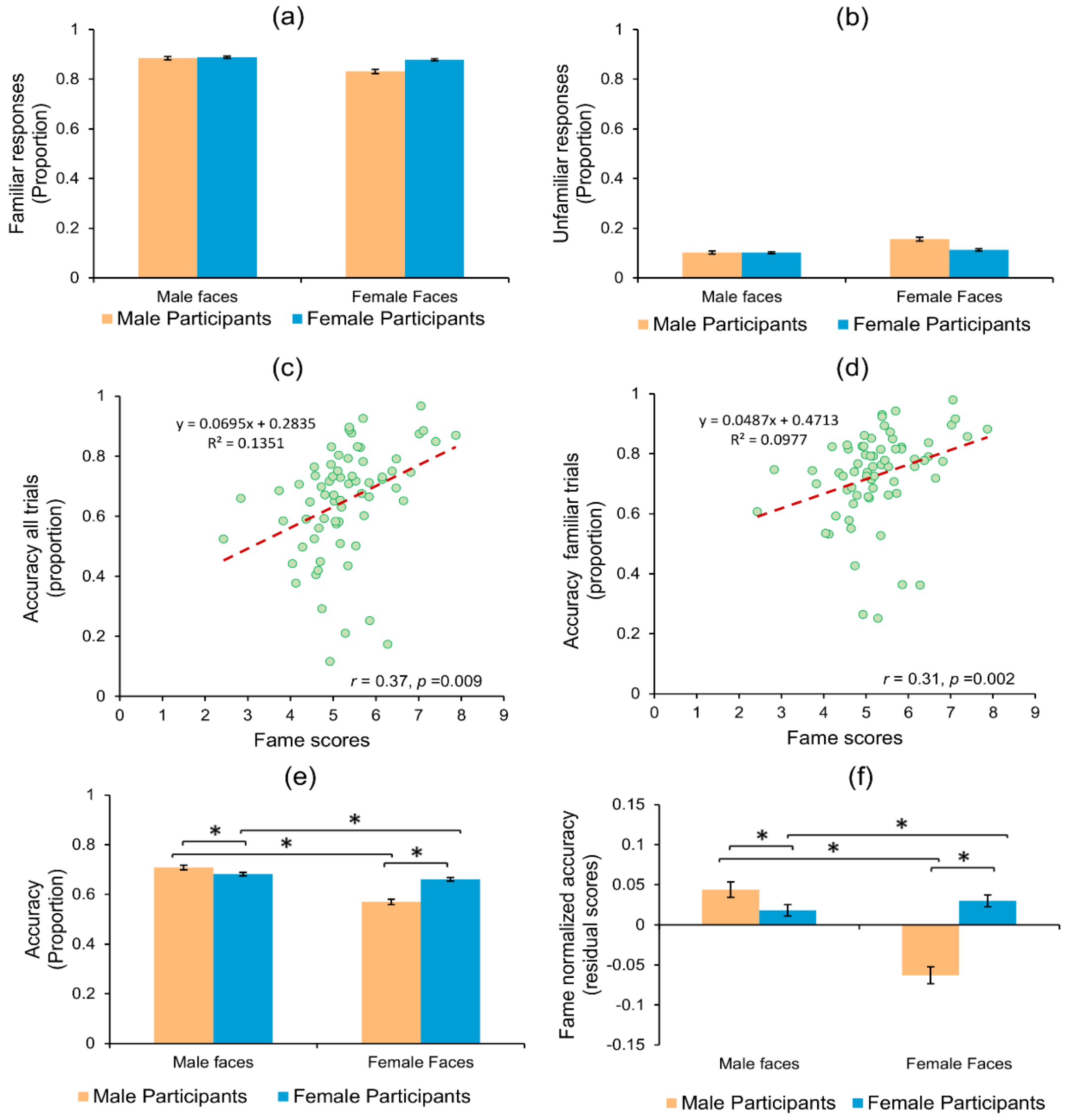

Figure S1. USA Dataset. Bar plots for a) Total proportion of familiar trials that were selected for main analysis (ref. Fig 2). b) Proportion of unfamiliar trials excluded from main analysis for each group of participant and face gender. Scatterplots show the linear fit trend line, correlation coefficient and equation used to calculate fame scores for c) all the trials and just for the (d) familiar trials. In the main analysis the equation from (d) panel was used to calculate the residual scores for USA sample data and fame normalize the accuracy values. Bar plots (e) and (f) show the data when all the trials (familiar and unfamiliar) were used to conduct the gender analysis, as like the main figures.

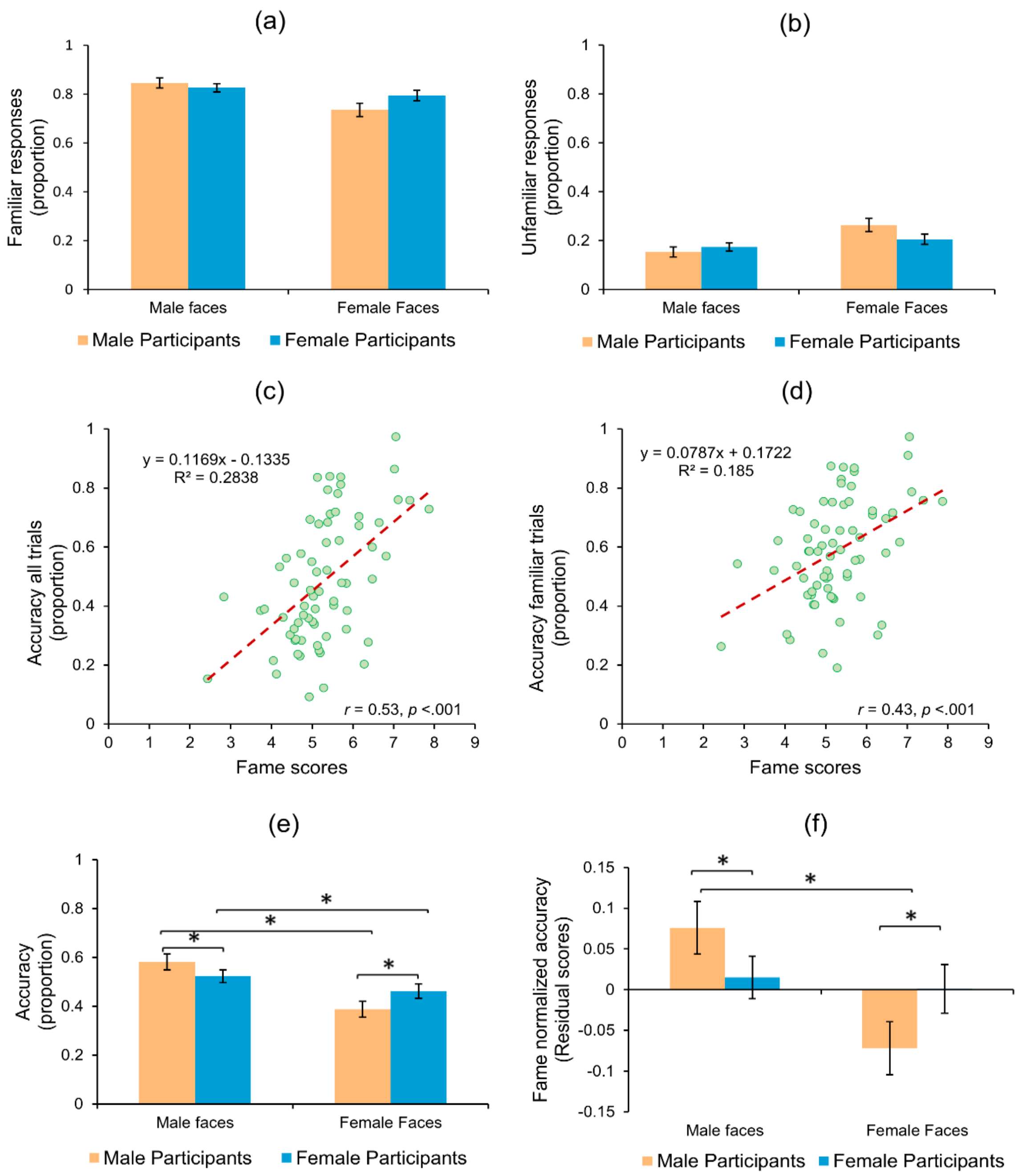

Figure S2. High Gender Equal countries dataset. Bar plots for a) Total proportion of familiar trials that were selected for main analysis (ref. Fig 3). b) Proportion of unfamiliar trials excluded from main analysis for each group of participant and face gender. Scatterplots show the linear fit trend line, correlation coefficient and equation used to calculate fame scores for c) all the trials and just for the (d) familiar trials. Bar plots (e) and (f) show the data when all the trials (familiar and unfamiliar) were used to conduct the gender analysis, as like the main figures.

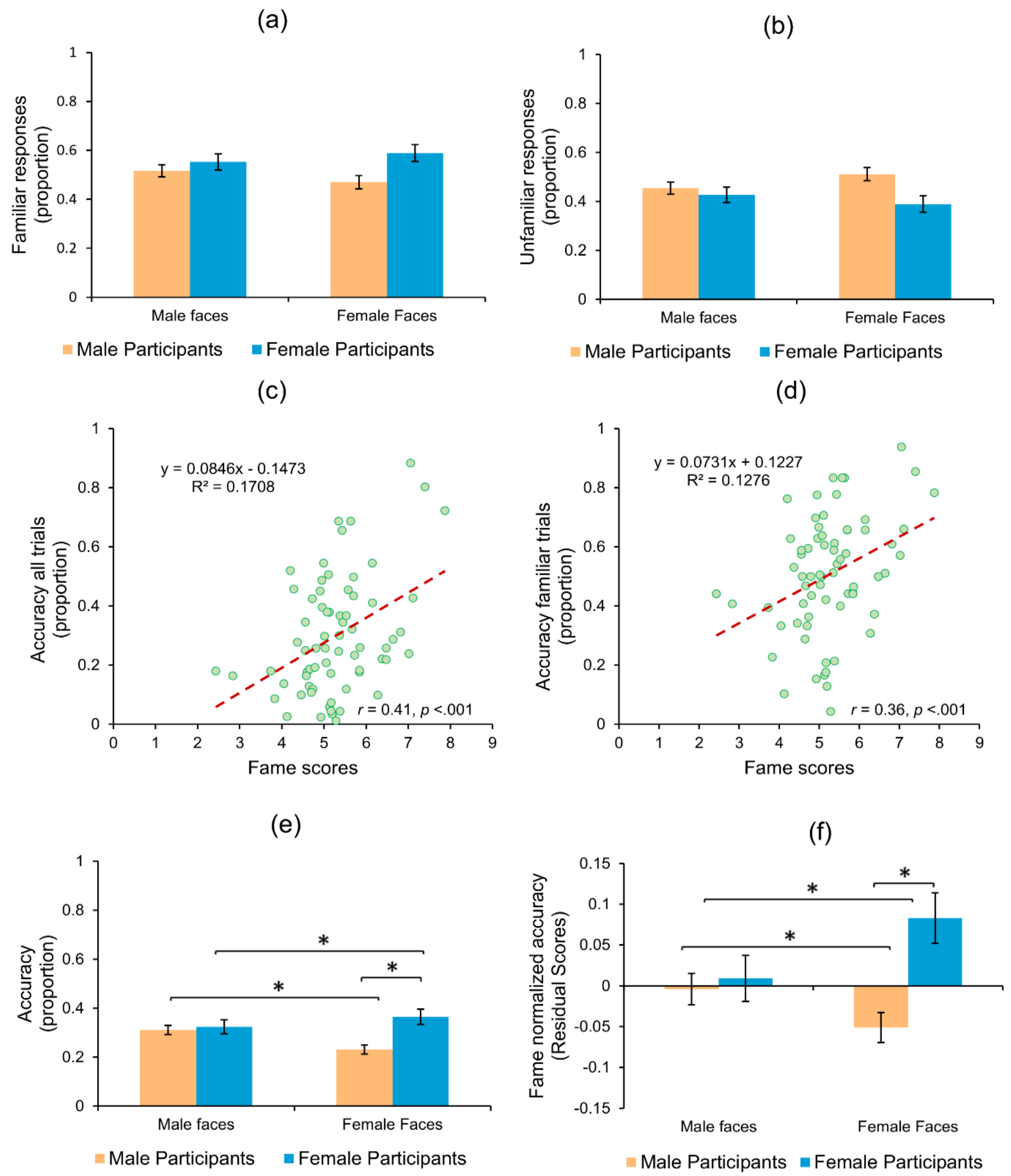

Figure S3. Low Gender Equal countries dataset. Bar plots for a) Total proportion of familiar trials that were selected for main analysis (ref. Fig 4). b) Proportion of unfamiliar trials excluded from main analysis for each group of participant and face gender. Scatterplots for c) all the trials and just for the (d) familiar trials. Bar plots (e) and (f) show the data when all the trials (familiar and unfamiliar) were used to conduct the gender analysis, as like the main figures.

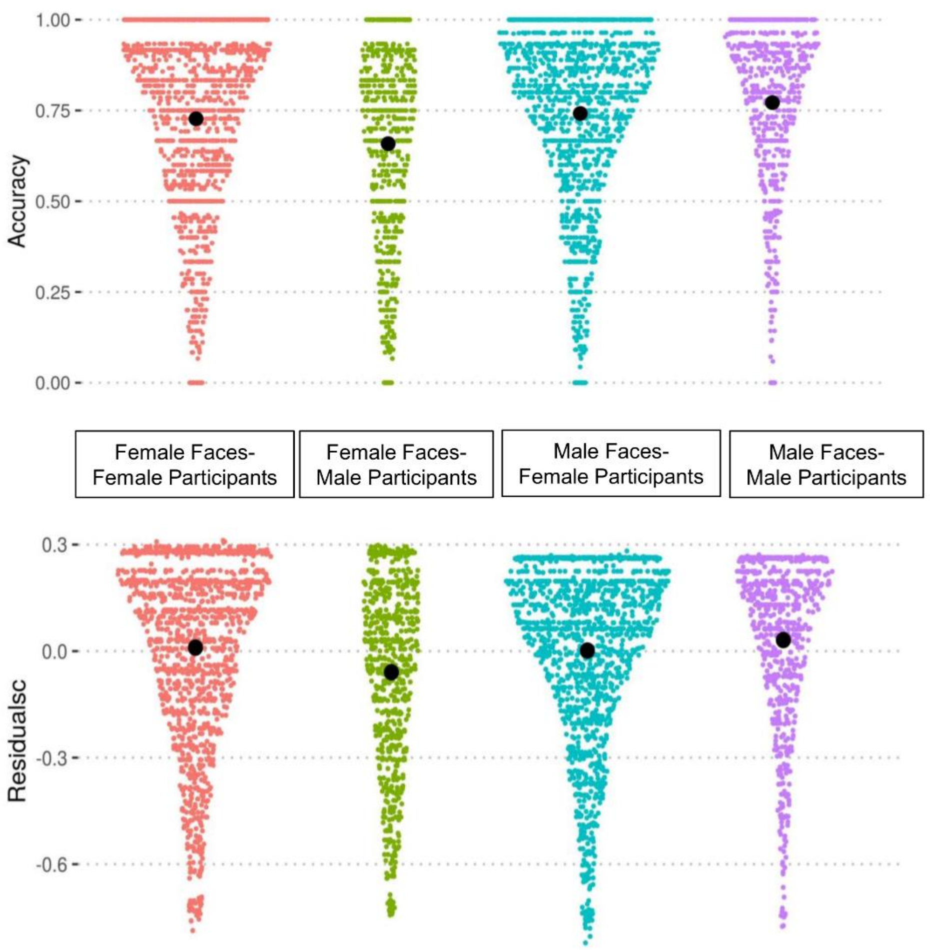

Figure S4. USA data distribution using sinaplots. Each dot represents individual participant raw accuracy (top row) and residual scores (bottom row) for familiar trials, for given gender of the faces. The length of the plot represents the score values while the width represents the number of participants with corresponding scores (N>2000). The black dot represents the mean of the distribution. These data were used for main statistical analysis. As can be seen, female participants responses were more widespread and distributed in comparison to males.

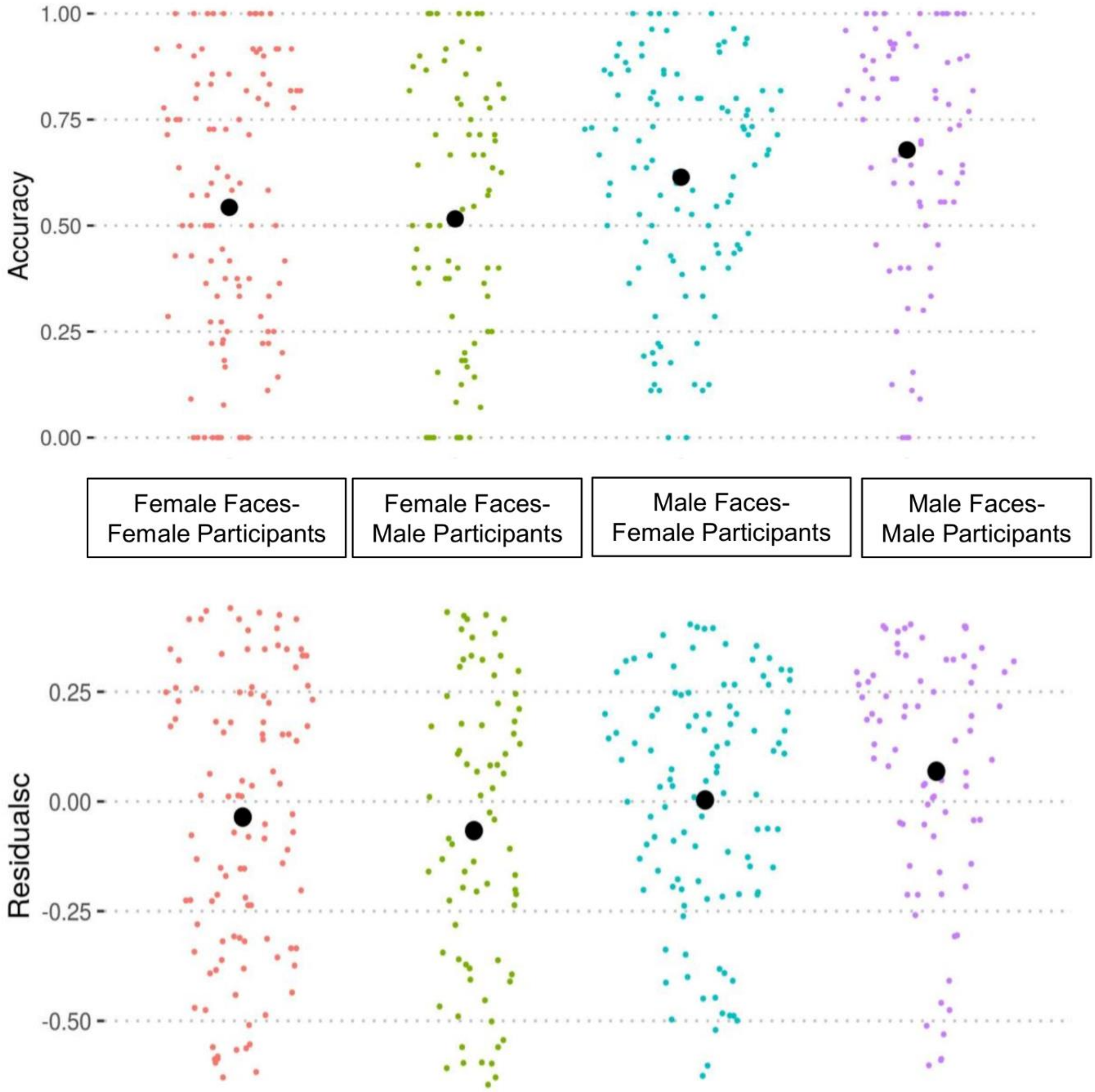

Figure S5. High Gender equal countries data distribution. Each dot represents individual participant raw accuracy (top row) and residual scores (bottom row) for familiar trials, for given gender of the faces. The length of the plot represents the score values while the width represents the number of participants with corresponding scores (N >200). The black dot represents the mean of the distribution. These data were used for main statistical analysis.

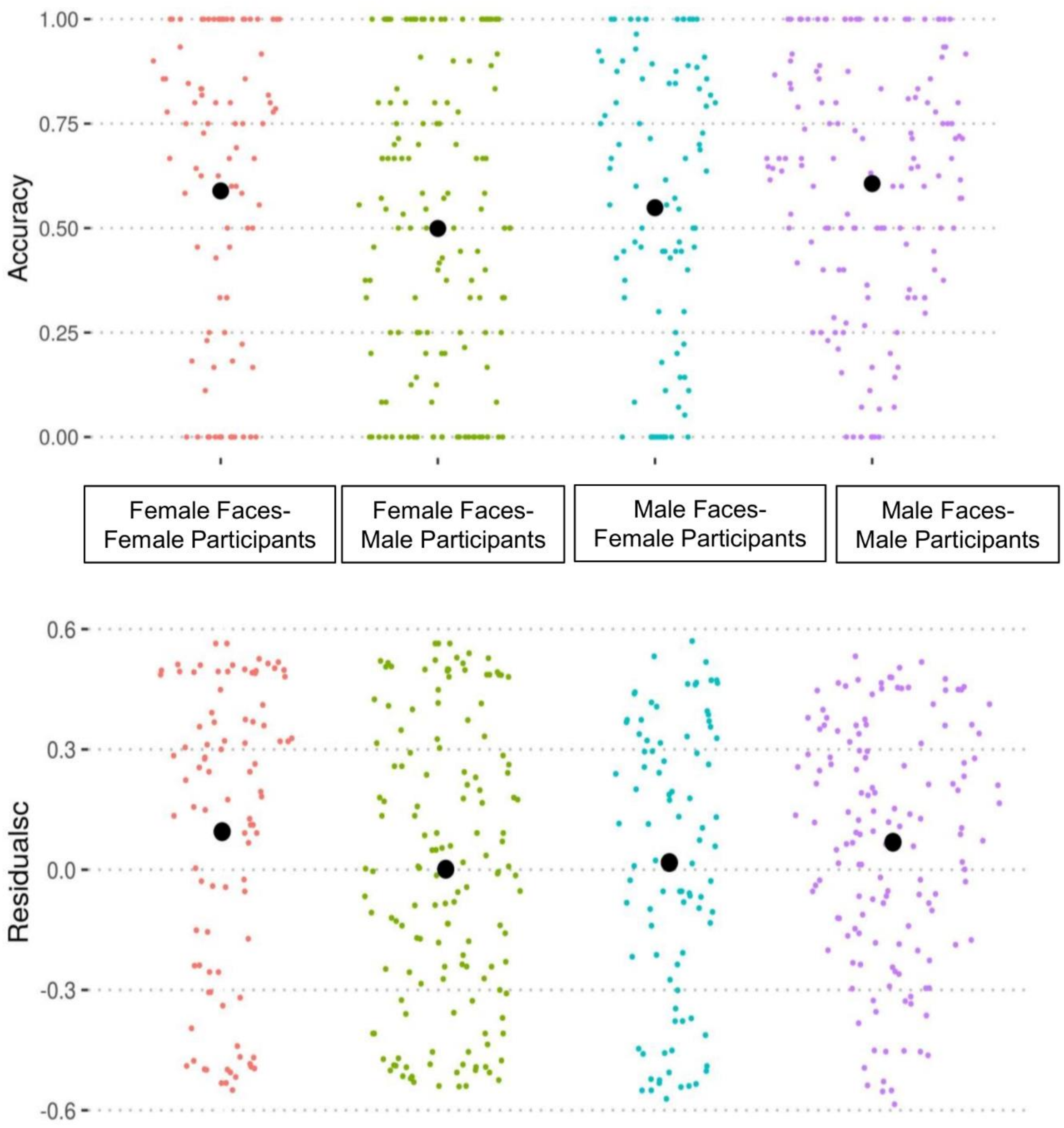

Figure S6. Low gender equal countries data distribution. Each dot represents individual participant raw accuracy (top row) and residual scores (bottom row) for familiar trials, for given gender of the faces. The length of the plot represents the score values while the width represents the number of participants with corresponding scores (N>200). The black dot represents the mean of the distribution. These data were used for main statistical analysis.

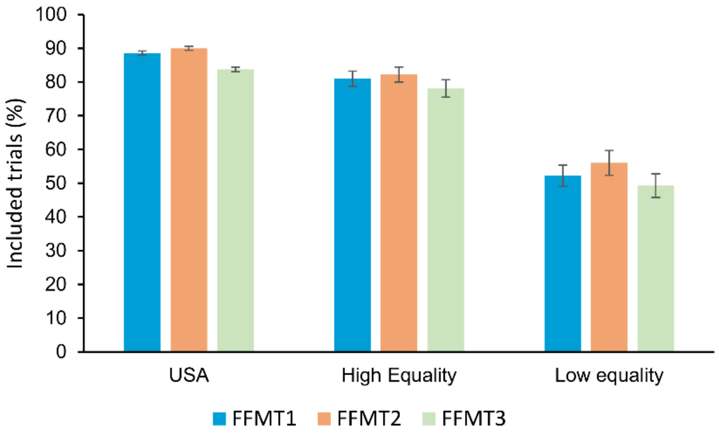

Figure S7. Trials included in each test. The figure shows the percentage of trials (familiar face trials) that were included in this study across the three different tests and country groups. Error bars represent standard error of mean.
